# Monoclonal Gammopathy of Renal Significance with Deposits of Infrequent Morphology: Two Case Reports of Light and Heavy Chain Deposition Disease with Atypical Presentation and Literature Review

**DOI:** 10.3390/medicines10100055

**Published:** 2023-10-04

**Authors:** José C. De La Flor, Maribel Monroy-Condori, Jacqueline Apaza-Chavez, Iván Arenas-Moncaleano, Francisco Díaz, Xavier E. Guerra-Torres, Jorge L. Morales-Montoya, Ana Lerma-Verdejo, Edna Sandoval, Daniel Villa, Coca-Mihaela Vieru

**Affiliations:** 1 Department of Nephrology, Hospital Central de la Defensa, 28046 Madrid, Spain; 2 Section of Nephrology and Hypertension, Hospital General Universitario Nuestra Señora del Prado, 45600 Talavera de la Reina, Spain; mmonroy@sescam.jccm.es (M.M.-C.); igarenas@sescam.jccm.es (I.A.-M.); xguerra@sescam.jccm.es (X.E.G.-T.); jmmontoya@sescam.jccm.es (J.L.M.-M.); 3 Department of Nephrology, Hospital Fuensanta, 28027 Madrid, Spain; japaza77@gmail.com; 4 Department of Anatomic Pathology, Hospital Gregorio Marañón, 28007 Madrid, Spain; fdiazc@salud.madrid.org (F.D.); vieru.mihaela26@gmail.com (C.-M.V.); 5 Department of Hematology, Hospital General Nuestra Señora del Prado, 45600 Talavera de la Reina, Spain; analermaverdejo@gmail.com; 6 Department of Hematology, Hospital Central de la Defensa, 28046 Madrid, Spain; esanba5@mde.es; 7 Department of Nephrology, Hospital Clínica Universiad Navarra, 31009 Pamplona, Spain; daniel.villa.hurtado@gmail.com

**Keywords:** monoclonal gammopathy of renal significance, monoclonal immunoglobulin deposits disease, light and heavy chain deposition disease

## Abstract

Background: Monoclonal immunoglobulin deposition disease (MIDD) includes three entities: light chain deposition disease (LCDD), heavy chain deposition disease (HCDD) and light and heavy chain deposition disease (LHCDD). The renal presentation can manifest with varying degrees of proteinuria and/or nephrotic syndrome, microhematuria, and often leads to end-stage renal disease. Given the rarity of LHCDD, therapeutic approaches for this condition remain inconclusive, as clinical trials are limited. Case presentation: We report two male patients with underlying monoclonal gammopathy of renal significance (MGRS) associated with LHCDD lesions. Both cases had non-nephrotic proteinuria, moderately impaired renal function, and normal levels of C3 and C4. Light microscopy of the renal biopsies in both patients did not show lesions of nodular glomerulosclerosis. Immunofluorescence showed a staining pattern with interrupted linear IgA-κ in patient #1 and IgA-λ in patient #2 only along the glomerular basement membrane (GBM). Electron microscopy of patient #1 revealed electrodense deposits in the subendothelial and mesangial areas only along the GBM. Discussion: In this case series, we discuss the clinical, analytical, and histopathological findings of two rare cases of LHCDD. Both patients exhibited IgA monoclonality and were diagnosed with monoclonal gammopathy of undetermined significance (MGUS) by the hematology department at the time of renal biopsy. Treatment with steroids and cytotoxic agents targeting the clone cells responsible for the deposition disease resulted in a favorable renal and hematologic response.

## 1. Introduction

Monoclonal gammopathy of renal significance (MGRS) is a hematologic disorder characterized by the proliferation of B lymphocyte or small plasma cell clones that produce and release a monoclonal immunoglobulin (MIg) or its components (light or heavy chains) in patients who do not meet the diagnostic criteria for multiple myeloma (MM) or other B-cell malignancies [1]. This paraprotein can affect multiple organs including the kidneys, leading to damage in the tubular, glomerular, vascular, or interstitial compartments through direct (deposition) or indirect (alterations of the alternative complement pathway) mechanisms, resulting in a heterogeneous group of entities. Renal diseases associated with MGRS exhibit distinct pathogenesis, clinical presentation, and renal biopsy findings. Loss of the glomerular filtration rate is a significant contributor to the morbidity in MGRS. Despite the absence of a high tumor burden, M protein plays a direct role in the pathogenesis of renal disease and is associated with an increased risk of progression to end-stage renal disease (ESRD) and recurrence after renal transplantation. Therefore, early diagnosis is crucial to stop the clonal production of immunoglobulins [1,2].

The histopathological classification, according to the consensus document of the International Kidney and Monoclonal Gammopathy Working Group (IKMG), is based on the characteristics of the MIg deposits identified using electron microscopy (EM): organized, non-organized, or absent deposits [3]. Within the non-organized deposits, we encounter monoclonal immunoglobulin deposition disease (MIDD), which is defined by the deposition of MIg along the glomerular and tubular basement membranes (GBMs, TBMs). Renal involvement is the most common manifestation in MIDD, followed by cardiac and hepatic disease. MIDD comprises three entities: light chain deposition disease (LCDD), heavy chain deposition disease (HCDD), and light and heavy chain deposition disease (LHCDD) [4]. LCDD is the most common form of MIDD, with a prevalence of approximately 80–90% of cases, while LHCDD and HCDD have similar frequencies, at 8% and 9%, respectively [2].

The histologic features of renal damage identified using light microscopy (LM) in MIDD, LCDD, LHCDD, and HCDD are similar. MIDD produces nodular lesions with a disease progression that mimics nodular diabetic glomerulosclerosis in 75–80% of cases [5]. The deposits of MIDD are negative upon Congo red staining. Immunofluorescence (IF) shows linear diffuse non-amyloid MIg staining in GBM and TBM for one of the light chains (LC) (kappa-κ or lambda-λ) in LCDD, a single LC (κ or λ) associated with a single heavy chain (HC) in LHCDD, or a single HC without an accompanying LC in HCDD. In LCDD, the most frequently observed component corresponds to a κ-LC, whereas deposits in HCDD and LHCDD usually correspond to γ(IgG) and γ(IgG)-κ, respectively [4]. Finally, the evidence of electrodense non-fibrillary deposits in the GBMs and TBMs is demonstrated using electron microscopy (EM).

Although the deposit of one type of LC or HC may vary, the clinical findings are identical among the three types of MIDD [6]. The renal presentation is common and can manifest with varying degrees of proteinuria and/or nephrotic syndrome, microhematuria, and often leads to ESRD [7,8]. Arterial hypertension (AHT) is present in 55% to 80% of patients [7]. Extrarenal manifestations have been reported in the heart, liver, and brain, predominantly affecting patients older than 50 years [7]. Clinical evidence of dysproteinemia is present in 97% of patients, and the most common associated hematologic conditions are MGRS (66%), MM (33%), Waldrenstrom’s macroglobulinemia (WM) (3%) and chronic lymphocytic leukemia (CLL) (1%) [2].

The treatment of the three types of MIDD remains ill-defined and is based on decreasing the production of pathologic MIg to prevent its deposition in tissues. The treatment recommendations are similar to MM, but it is usually less effective than MM and can cause considerable side effects [8]. To improve the morbidity and mortality of these patients, the current therapeutic consensus suggests a clone-directed approach, using antimyeloma agents with low toxicity, such as bortezomib, lenalidomide, and daratumumab in patients with plasma cell clones, and rituximab-containing regimens in patients with lymphocytes or lymphoplasmacytic clones [9]. If there are no contraindications, induction therapy is complemented by autologous transplantation with the intention of slowing down the continued deposition of LC/HC and stabilizing or even improving the function of the affected organs [9,10]. During follow-up as well as at diagnosis, it is recommended to evaluate the degree of proteinuria. It is well known that proteinuria is an established marker of kidney damage as well as a risk factor for the progression of ESRD. Flammia et al. [11] reported preoperative proteinuria as a significant predictor of lower overall survival and worse renal functional outcomes in patients undergoing renal cancer surgery.

LHCDD is a rare systemic disease and an unusual presentation of MGRS. Herein, we report two cases of LHCDD as an expression of MGRS, both of which showed good renal and hematologic responses to induction treatment.

## 2. Case Presentation

**Case 1**

The first case is a 78-year-old male with a history of well-controlled type 2 diabetes mellitus (T2DM) (glycated hemoglobin-HbA1c of 5.2%), AHT, dyslipidemia, and chronic kidney disease (CKD) stage 3aA1 with serum creatinine (SCr) and a urinary albumin/creatinine ratio (UACR) of 1.25 mg/dL and 70 mg/g, respectively. His medication included angiotensin II receptor blockers (ARBs), sodium-glucose co-transporter 2 inhibitors (SGLT2i), metformin, mineralocorticoid-receptor antagonists (MRA), and statins. In October 2020, he was referred to our outpatient clinic due to a significant increase in proteinuria, with a UACR of 745 mg/g. A physical examination revealed a blood pressure of 130/80 mmHg, a respiratory rate of 24 breaths/min, and an oxygen saturation of 96% while breathing room air, without pulmonary rales or peripheral edema. There was no lymph node involvement, splenomegaly, arthralgia, bone pain, or Raynaud’s phenomenon. Complementary tests showed the following: a hemoglobin level of 13 g/dL, a platelet count of 143 × 10^9^/L, an LDH of 385 U/L, normal haptoglobin, reticulocytes at 1.26%, a blood smear without schistocytes, a negative Coombs test, a negative rheumatoid factor (RF), and normal ADAMTS-13. There was no hypoalbuminemia or hypercholesterolemia. The 24 h urine protein excretion was 1.59 g, with a spot urine protein-to-creatinine ratio (UPCR) and UACR of 1200 and 645 mg/g, respectively. Serum protein electrophoresis (SPEP) and serum immunofixation electrophoresis (SIFE) revealed a monoclonal band IgA-κ. Urine protein electrophoresis (UPEP) and urine immunofixation electrophoresis (UIFE) were positive for IgA-κ. Serum free light chain kappa (FLCκ) and lambda (FLCλ) were 31.2 mg/dL and 12 mg/L, respectively, with a κ/λ FLC ratio of 2.61. The complement C3 and C4 studies were normal. The rest of the immunological and viral studies were negative (shown in Table 1). Abdominal sonography revealed normal-sized kidneys.

A bone marrow (BM) biopsy revealed 6% plasma cells, with negative Congo red staining. The immunophenotypic study showed that 3.1% of the total cellularity comprised plasma cells, and of these, 89% had an abnormal phenotype with monoclonality for the kappa light chain. However, the identi-clone assay was not performed. A positron emission tomography-computed tomography scan (PET/CT) did not suggest lymphoproliferative syndrome, solitary plasmacytoma, or other extramedullary involvements. The bone series study showed no evidence of lytic or blastic lesions.

**Figure 1:**
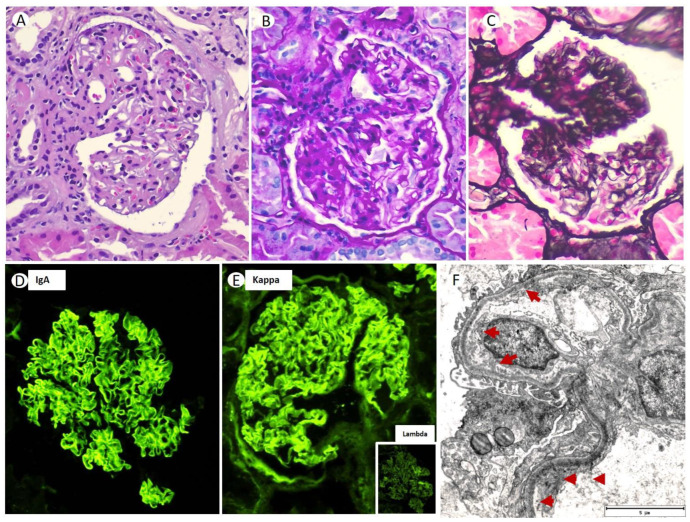
Glomerulus showing mesangial matrix expansion without an increase in mesangial cellularity ((**A**) original magnification ×400; hematoxylin-eosin stain). Segmental sclerosis of the capillary tuft observed with periodic acid Schiff (PAS) techniques ((**B**) original magnification ×400) and silver-methenamine staining ((**C**) original magnification × 400). Direct immunofluorescence demonstrated diffuse linear GBM staining for IgA (3+) (**D**), with restriction for kappa (++)/lambda (−) light chains (inner-box) (**E**) (×40). Electron microscopy showed fine granular subendothelial deposits of the Randall type (**F**) (×2000).

Thus, a renal biopsy (RB) was performed; 35 glomeruli could be analyzed. Among them, five (14.2%) were globally sclerotic. Under LM, the glomeruli showed a markedly expanded mesangium at the expense of the matrix, without mesangial hypercellularity (Figure 1A). The capillary loops were thickened, with permeable capillary lumens, without endocapillary hypercellularity or intracapillary thrombi. No extracapillary proliferation was observed. In two glomeruli (5.7%), lesions of segmental sclerosis of the capillary tangle were identified (Figure 1B,C). Silver staining did not identify thrombi, spicular projections, or double contour images in the glomerular capillaries (Figure 1C). Congo red and thioflavin staining were negative. There was mild inflammatory interstitial fibrosis, predominantly lymphoplasmacytic, associated with a focus of minimal tubular atrophy and focal fibrosis (less than 10%). Routine frozen tissue immunofluorescence (IF-F) performed on seven glomeruli showed diffuse and linear staining for IgA (2+) with kappa (2+) LC restriction along the GBM (Figure 1D,E). The rest of the antisera were negative (IgG, IgM, C1q, C3, C4, lambda, and fibrinogen). Detection of IgA subclasses was not performed. The IF on pronase-digested paraffin-embedded (IF-P) sections showed similar results to that obtained via IF-F. EM showed electron-dense non-organized granular deposits in the subendothelial, mesangial area, and along the segmental GBM (Figure 1F). Based on these LM, IF, and EM findings, a final diagnosis of MGRS with lesions of LHCDD IgA-kappa superimposed with diabetic glomerulosclerosis class IIb was suspected.

In view of the above findings, treatment with bortezomib and dexamethasone (BordD) chemotherapy was initiated. Bortezomib was administered at a dose of 1.3 mg/m^2^ given as an SC bolus on days 1, 8 and 15, with dexamethasone 20 mg PO once weekly on days 1, 8, 15 and 22 of the Bortezomib treatment cycle. The patient completed eight cycles of induction. After six cycles of BordD treatment, total renal response was achieved, defined as the SCr decreasing to less than 1 mg/dL and a decrease in proteinuria by >50% to 0.3 g/24 h. Additionally, a partial hematological response was achieved, with a reduction of more than 50% in kappa light chain levels.

**Case 2**

The second case is a 57-year-old Caucasian male with a previous medical history of high blood pressure, dyslipidemia, and monoclonal gammopathy of undetermined significance (MGUS) IgA lambda, under the care of a hematologist. The patient’s medication includes ARB, hydrochlorothiazide, calcium receptor antagonists, and a statin.

The BM biopsy revealed less than 10% plasma cells, with negative Congo red staining. The immunophenotypic study showed that 3% of the total cellularity comprised plasma cells, and of these, 98% exhibited an immunophenotype compatible with MM: CD38++, CD138++, CD45+d, CD19−, CD56+, lambda light chain restriction, CD81−, CD28−, CD 27++, and CD117−. The subcutaneous fat biopsy showed negative Congo red staining, and the echocardiogram was normal. A PET/CT scan revealed no evidence of lymphoproliferative syndrome, solitary plasmacytoma, or other extramedullary manifestations.

The patient was referred to the nephrology department for the evaluation of suspected MGRS. Laboratory findings revealed a serum creatinine (SCr) level of 1.19 mg/dL, an estimated glomerular filtration rate (eGFR) of 68 mL/min/1.73m^2^ calculated via the Chronic Kidney Disease Epidemiology (CKD-EPI) formula, and normal serum electrolyte levels. The 24 h urine protein excretion was 1.2 g, while SPEP/SIFE and UPEP/UIFE showed monoclonal bands of IgA lambda with positive Bence Jones protein. The patient’s serum IgA level was elevated at 1510 mg/dL, with normal levels for IgG and IgM. The rest of the immunological tests were normal and viral studies were negative (shown in Table 1).

**Figure 2:**
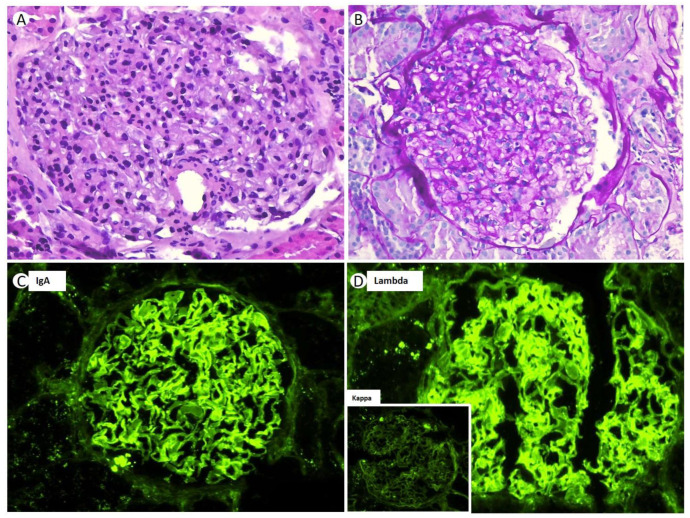
Glomerulus showing mesangial matrix expansion with increase in mesangial cellularity ((**A**) original magnification, ×400; hematoxylin-eosin stain). PAS staining showing the same characteristics ((**B**) original magnification, ×400). Direct immunofluorescence shows a global and diffuse linear deposit in the glomerular capillary loops for IgA (3+) (**C**), and with lambda light chain (LC) restriction (2+), there is no deposition of kappa LC (inner-box) (×40) (**D**).

After performing an RB, 26 glomeruli were analyzed, of which four were globally sclerotic (15%). Under LM, the renal parenchyma presented a preserved general architecture. The glomeruli displayed a hypertrophic and congestive aspect, with slight expansion of the mesangial matrix, associated with focal mesangial hypercellularity (Figure 2A). The expanded mesangial matrix showed the usual staining characteristics with histochemical techniques, being positive when using the periodic acid Schiff (PAS) and silver-methenamine (SM) techniques (Figure 2B). The capillary lumens were permeable, and focal endocapillary hypercellularity was observed to be primarily composed of neutrophilic polymorphonuclear cells (Figure 2A). No spicular projections were observed in the subepithelial aspect of the GBM, nor were double contour images, although there were focal vacuolar changes in the GBM. The interstitium showed minimal fibrosis (less than 10%), without inflammatory infiltrates. The tubules did not present any significant histologic changes, regenerative changes, intracytoplasmic or intraluminal crystalloid morphology structures, nor tubulitis. The arterioles had a preserved appearance, and no intravascular thrombi or vasculitis were observed. The IF-F, performed on nine glomeruli, showed a global and diffuse linear pattern for IgA (2+) and lambda (2+) in glomerular capillary loops and GBM (Figure 2C,D). The patient also presented positivity for albumin (++) in GBM, without the presence of deposits for the rest of the antisera used (IgG, IgM, C1q, C3, C4, Kappa, and fibrinogen). The IF-P sections showed similar results to those obtained using IF-F. Immunohistochemistry techniques were positive for IgA and lambda in glomerular capillary loops and negative for kappa and C4d. The Congo red stain was negative.

Based on the above findings, a diagnosis of MGRS with lesions of LHCDD IgA-lambda was performed. The patient underwent treatment with bortezomib, lenalidomide, and dexamethasone chemotherapy. Bortezomib was administered at a dose of 1.7 mg/m^2^ given as an SC bolus on days 1, 8 and 15, with dexamethasone 40 mg PO once weekly on days 1, 8, 15 and 22 of the bortezomib treatment cycle. Lenalidomide was administered at a dose of 10 mg PO on days 1 through 28 of the repeated 28-day cycles. Patient completed eight cycles of induction. The treatment was well tolerated by the patient, achieving total renal and hematologic remission.

## 3. Discussion

We present two male patients with monoclonal IgA-κ and IgA-λ underlying MGRS associated with LHCDD lesions. According to the 2019 IKMG consensus document, MIDD was included in the histological classification of non-organized deposits as part of the MGSR lesions [3]. Previously, the term MIDD, historically referred to as Randall-type (amorphous or granular and non-Congophilic) MIDD, was used to encompass a wide variety of paraprotein-related diseases, including light chain amyloid (LCA) and myeloma light chain cast nephropathy (LCCN). However, after the introduction of the term MGSR, the majority of MIDD cases have been linked to plasma cell dyscrasia. MIDD is relatively rare, with a pure incidence, excluding cases of LCCN or LCA in native kidneys, ranging from 0.32 to 0.7% according to the described series [5,7]. On the other hand, MM or smoldering MM are present in almost 50% of LHCDD cases [6,12], while the combination of LCDD and LCCN is the most commonly described, accounting for 58–65% of cases [5,7,13]. Many authors have considered LHCDD as a variant of LCDD, and although information on the clinicopathologic features and prognosis of LHCDD is limited, there are some relevant differences between them. LHCDD is the least frequent entity of MIDD and its association with MGRS is unusual [2]. LHCDD is the term used when LCDD and HCDD cases are combined, with γ HC and κ LC being the most frequent components in these patients [5]. LHCDD is a systemic disease with deposition of abnormal LC and HC in a variety of organs, but deposition in the renal parenchyma is the most frequently described and most often leads to clinical dysfunction [2,5].

Clinically, differentiating the three types of MIDD (LCDD, HCDD, and LHCDD) from MM-LCCN and amyloidosis is crucial. While amyloidosis typically presents as a nephrotic syndrome with extensive extra-renal manifestations, MIDDs exhibit greater variability, including various degrees of proteinuria, microhematuria, and renal failure, and MIDD has a greater tendency to be limited to the kidney. Our two cases demonstrated non-nephrotic proteinuria, moderately impaired renal function, and normal complement C3 and C4 levels. Microhematuria was observed in only one patient. Both patients had IgA monoclonality and were initially diagnosed as MGUS at the time of RB. However, unlike our patients, a clinical-pathologic series of MIDD by Nasr et al. [5] reported six cases with LHCDD lesions. These patients had proteinuria in the nephrotic range, high blood pressure, edema, nephrotic syndrome, and leukocyturia. Notably, early diagnosis of LHCDD in our series might account for these discrepancies. The clinical tests for dysproteinemia were positive for both patients via SPEP/SIFE and UPEP/UIFE. While IgG (67%) and κ LC (71%) were the most frequent types of serum monoclonal immunoglobulins and light chains in Nasr’s series [5], our cases showed monoclonal IgA with abnormal κ and λ LC quantification, not meeting the criteria for MM. Based on the underlying hematologic disease (MGUS) and the RB findings, the patients were diagnosed with MGRS featuring LHCDD lesions. Although hypocomplementemia is common in LHCDD patients, likely due to the activation of the classical complement pathway, particularly subclass γ (IgG1 and IgG3), it was not observed in our patients, possibly attributed to the weak complement system activation of IgG2. However, this assumption warrants further investigation, as we lack laboratory techniques for determining Ig subclasses in our hospital.

Histologically, the LM in pure MIDD (LCDD, HCDD, or LHCDD) presents diverse morphologic patterns resulting from glomerular proliferation and mesangial matrix expansion. Nodular sclerosing glomerulopathy is a frequently observed lesion in the advanced stages, as reported in earlier reports [7], including Nasr’s series [5]. Notably, nodular mesangial sclerosis occurs more frequently in HCDD than in LCDD or LHCDD [5,9]. The early stages of the disease may exhibit focal and segmental mesangial nodules that worsen over time, leading to mesangial nodularity and variable thickening of the peripheral capillary wall. These mesangial nodules stain positively for PAS and trichrome red-blue trichrome, but negatively for Congo red, resembling diabetic nephropathy (DN). Ronco et al. [14] described some similarities between MIDD and DN in LM, including: (1) the nodules in LCDD, LHCDD and HCDD being acellular; (2) significant mesangial proliferation being present in LHCDD; (3) the nodules being PAS-positive and Congo red-negative; and (4) the GBMs appearing to be focally thickened with or without double contours. However, some differences have been reported to help discriminate between one disease or another; the nodules in MIDD tend to be uniform in size, whereas in DN, they are generally irregular in distribution, and vary in size and shape within given glomeruli and among glomeruli. The LM of the RB of our two patients did not show lesions of nodular glomerulosclerosis; only one patient had a long history of controlled T2DM. Patient #1 showed a markedly expanded mesangium without mesangial hypercellularity, while patient #2 showed a light expansion of the mesangial matrix with focal mesangial hypercellularity. In addition, it is also necessary to perform the differential diagnosis with other entities such as amyloidosis, membranoproliferative glomerulonephritis (MPGN), immunotactoid glomerulopathy (ITGN) and fibrillary glomerulonephritis (FGN).

However, definitive diagnosis cannot be performed with LM alone, and IF is critical for the diagnosis of three types of MIDD. IF allows for the subclassification of MIDD cases, depending on the staining profile of the monoclonal protein. In cases of LHCDD, a diffuse linear pattern of monotypic LC (usually κ) associated with one of the HCs (usually γ) along the GBM and TBM can usually be observed [5,7]. Especially, TBM deposits are strongly stained, whereas staining in GBM is weak when using IF [14]. The five cases of LHCDD of the series reported by Lin et al. [7] included three with IgG-κ and two with IgG-λ. Masai et al. [15] identified six patients with LHCDD from 5433 RBs, three patients had IgG-k deposits and three patients had IgG- λ deposits. Heavy chain subclass analysis performed in four patients showed IgG3 deposits in all patients, while in Nasr’s series [5], the six patients with LHCDD showed three IgG-κ, one IgA-κ, and one IgA-λ. It is notable to mention that in our series, IF of two patients showed interrupted linear patterns of κ in patient #1 and λ in patient #2 as LC, and both IgA as HC along the GBM, but not along the TBM. Additional IF-P sections of both cases showed no staining for LC and HC along the TBM.

In addition, EM helps us to provide confirmatory evidence of LC and HC deposits in the various renal compartments. EM of MIDD cases showed deposition of electrodense, granular, punctate, non-fibrillar deposits within all renal basement membranes. The GBM deposits are concentrated along the inner aspect of the lamina dense and subendothelial zone, extending to the mesangial matrix, while that TBM deposits are located along the outer aspect of the TBM and in the interstitium proper [6]. These deposits also are observed in the arterioles’ intima and BMs, or interstitial capillary BMs [6]. In our series, we only obtained a specimen for EM in patient #1, which showed deposits of electrodense material in the sub-endothelial, mesangial area and along the GBM, with no electrodense deposits along the TBM. Nakamura et al. [16] reported similar data of a case concurrence of LHCDD and diabetic nodular glomerulosclerosis based on GBM deposits via IF and EM. In addition, Masai et al. [15] showed that among a series of six LHCDD patients, no TBM deposits were identified in one patient. However, in the longest series of MIDD patients, for the five patients with LHCDD reported by Lin et al. [7], TBM deposits were detected in all cases. Meanwhile, in the six patients reported by Nasr et al. [5], electrodense deposits along the GBM and in mesangial areas were detected in all cases, and deposits along the TBM were only detected in five of them. On the other hand, Satirapoj et al. [17] reported a case of LHCDD and concurrent diabetic nodular glomerulosclerosis, and the authors revealed kappa and IgG deposits only along the TBM, and not in the GBM. We do not have a clear explanation for the absence of electrodense deposits in TBM as observed via ME, and negative staining for LC and HC along the TBM as observed via IF. However, this is not a reason not to diagnose LHCDD in our series.

Therefore, after ruling out LCA due to the negativity of the Congo red stain, and after ruling out a FGN and ITGN due to the presence of non-organized deposits in EM, we arrive at the final diagnosis of LHCDD IgA-κ in case #1 and IgA-λ in case #2. These cases represent a rare form of MGSR with an unknown prevalence.

Due to the rarity of LHCDD to date, the therapeutic approaches for this pathology are not consensual, as clinical trials are sparse. The main goal of treatment is to slow the production and tissue deposition of LC and HC to prevent further damage to the organ function. The treatment scheme used for MGRS should be considered the gold-standard treatment for LHCDD [18]. First-line chemotherapy treatment is based on high doses of dexamethasone and bortezomib, usually in the form of triple therapy, associated with immunomodulatory drugs such as lenalidomide, although its use is limited due to renal elimination. Regimens with bortezomib together with cyclophosphamide and dexamethasone are considered the first option, mainly in patients with renal failure. Other regimens with immunomodulators such as daratumumab are reserved for refractory or relapsed cases. The hematologist should consider the possibility of transplanting hematopoietic precursors after intense chemotherapy treatment. The treatment approach is based on chemotherapy, which needs to be tailored to the specific nature of the cell clone, either lymphocytic or plasma, renal function, and the presence or absence of extrarenal involvement [9,19]. In our two patients with LHCDD, the mean follow-up time was 12 months. Treatment with steroids and cytotoxic agents targeting the clone cell responsible for the deposition disease was effective, leading to a good renal and hematologic response.

## 4. Conclusions

In conclusion, we have presented two exceptional cases of MGRS featuring rare lesions of LHCDD. IF and IF-P sections of two patients showed diffuse linear patterns of κ in patient #1 and λ in patient #2 as LC, and both IgA as HC only along the GBM, but not in the TBM, as well as the absence of electrodense deposits in TBM as observed via ME in patient #1. LHCDD remains a rare disease, with an unknown prevalence, and its pathophysiology is not yet fully understood.

Accurate histopathologic identification plays a pivotal role in determining the optimal treatment approach. It is crucial for patients with MGRS to undergo comprehensive evaluation by a multidisciplinary team comprising nephrologists, hematologists, and nephropathologists to elucidate the causative role of the M-protein in renal disease pathogenesis. Currently, clone-directed therapy stands out as the most effective treatment strategy for these cases.

## Figures and Tables

**Table 1:** Laboratory findings on admission.

	Case 1	Case 2	Reference Range/Unit
WBC	9570	7300	U/L
Hemoglobin (Hb)	13	15.7	12–16 g/dL
Platelet count (PLt)	143	325	10^3^/μL
Reticulocytes count	1.26	NA	2–4%
Erythrocyte count	3.56	4.79	4.2–5.8 10^6^/μL
Lactate dehydrogenase (LDH)	155	260	228–428 IU/L
Coombs Test	Negative	NA	NA
Total Bilirubin	0.26	0.9	0.1–1 mg/dL
Total protein (TP)	6.9	7.4	6.4–8.7 g/dL
Serum Albumin (sAlb)	3.61	4.3	3–5.5 g/dL
GOT	18	33	2–41 IU/L
GPT	13	35	2–37 IU/L
Urea	51	36	17–60 mg/dL
Serum Creatinine	1.36	1,19	0.6–1.2 mg/dL
Na	141	138	135–145 mmol/L
K	4.7	4,0	3.5–5.5 mmol/L
Cl	103	105	95–110 mmol/L
CRP	1.5	3.0	0.1–0.5 mg/dL
Hbs-Ag	Negative	Negative	NA
HCV-Ab	Negative	Negative	NA
HIV	Negative	Negative	NA
C3 nephritic factor (C3NF)	Negative	Negative	Ratio > 1.022
C3	101	152	90–180 mg/dL
C4	34.1	24.1	10–40 mg/dL
RF	Negative	Negative	<15 IU/ml
ANA, Antids-DNA, ANCA and cryoglobulin	Negative	Negative	NA
Anti-GBM	Negative	NA	<1 AI
Anti-PLA2R Ab (ELISA)	Negative	Negative	NA
Beta 2 microglobulin	0.43	2.04	<0–20 mg/dL
IgG	500	460	800–1600 mg/dL
IgA	886	1510	70–400 mg/dL
IgM	20	43	90–180 mg/dL
UPCR	1200	421	<20 mg/g
UACR	645	294	<30 mg/g
Urine red blood cells	1–5	Negative	/HPF
24 h urine total protein excretion	1.59	1.2	<0.15 g/24-h
SPEP M-protein concentration	6%	1.16%	NA
SIFE	IgA kappa	IgA Lambda	NA
UPEP/UIFE	IgA kappa	IgA Lambda	NA
FLC κ	31.2	8.18	4.90–13.70 mg/L
FLC λ	12	47.5	7.60–19.50 mg/L
FLC κ/λ	2.6	0.17	0.27–1.67

AI: activity index, AU: arbitrary units, NA: not applicable, WBC: White blood cells, GOT: glutamate-oxaloacetate transaminase, GPT: glutamate pyruvate transaminase, Na: serum sodium, K: serum potassium, Cl: serum chloride, CRP: C—reactive protein, CH50: complement hemolytic activity-50, CFH: complement factor H, C3NF: complement 3 nephritic factor, C3: complement 3, C4: complement 4, RF: rheumatoid factor, ANA: antinuclear antibody, Antids-DNA: anti-double stranded DNA antibody, ANCA: anti-neutrophil cytoplasmic autoantibody, Anti-GBM: anti-glomerular basement membrane, Anti-PLA2R Ab: anti-phospholipase A2 receptor antibody, Ig: immunoglobulin, UPCR: spot urine protein-to-creatinine ratio, UACR: spot urine albumin-to-creatinine ratio, ELISA: Enzyme-Linked Immuno-Sorbent Assay, SPEP: serum protein electrophoresis, SIFE: serum immunofixation electrophoresis, UPEP/UIFE: urine protein electrophoresis/urine immunofixation electrophoresis, FLC: free light chain, κ: kappa, λ: lambda, HPF: high-power field.

## Data Availability

No new data were created or analyzed in this study. The data used to support the findings of this study are available from the corresponding author on request (Contact J.C.D.L.F., josedelaflor81@yahoo.com, jflomer@mde.es). I confirm that all the figures and tables are the original work of this manuscript’s authors. All have been created by the authors of this manuscript, have not been adapted from other authors, and do not present an online link.
